# Caspase-Dependent Inhibition of Mousepox Replication by gzmB

**DOI:** 10.1371/journal.pone.0007512

**Published:** 2009-10-19

**Authors:** Julián Pardo, Eva Ma Gálvez, Aulikki Koskinen, Markus M. Simon, Mario Lobigs, Matthias Regner, Arno Müllbacher

**Affiliations:** 1 Departamento Bioquímica y Biología Molecular y Celular, Facultad de Ciencias, Universidad de Zaragoza, Zaragoza, Spain; 2 Fundación Aragón I+D (ARAID), Gobierno de Aragón, Spain; 3 Instituto de Carboquímica, CSIC, Zaragoza, Spain; 4 Viral Immunology Group, Division of Immunology and Genetics, The John Curtin School of Medical Research, Australian National University, Canberra, Australian Capital Territory, Australia; 5 Molecular Virology Group, Division of Immunology and Genetics, The John Curtin School of Medical Research, Australian National University, Canberra, Australian Capital Territory, Australia; 6 Metschnikoff Laboratory, Max-Planck Institute for Immunobiology, Freiburg, Germany; Technical University Munich, Germany

## Abstract

**Background:**

Ectromelia virus is a natural mouse pathogen, causing mousepox. The cytotoxic T (Tc) cell granule serine-protease, granzyme B, is important for its control, but the underlying mechanism is unknown. Using *ex vivo* virus immune Tc cells, we have previously shown that granzyme B is able to activate several independent pro-apoptotic pathways, including those mediated by Bid/Bak/Bax and caspases-3/-7, in target cells pulsed with Tc cell determinants.

**Methods and Findings:**

Here we analysed the physiological relevance of those pro-apoptotic pathways in ectromelia infection, by incubating ectromelia-immune *ex vivo* Tc cells from granzyme A deficient (GzmB^+^ Tc cells) or granzyme A and granzyme B deficient (GzmA×B^−/−^ Tc cell) mice with ectromelia-infected target cells. We found that gzmB-induced apoptosis was totally blocked in ectromelia infected or peptide pulsed cells lacking caspases-3/-7. However ectromelia inhibited only partially apoptosis in cells deficient for Bid/Bak/Bax and not at all when both pathways were operative suggesting that the virus is able to interfere with apoptosis induced by gzmB in case not all pathways are activated. Importantly, inhibition of viral replication *in vitro*, as seen with wild type cells, was not affected by the lack of Bid/Bak/Bax but was significantly reduced in caspase-3/-7-deficient cells. Both caspase dependent processes were strictly dependent on gzmB, since Tc cells, lacking both gzms, neither induced apoptosis nor reduced viral titers.

**Significance:**

Out findings present the first evidence on the biological importance of the independent gzmB-inducible pro-apoptotic pathways in a physiological relevant virus infection model.

## Introduction

Cytotoxic T (Tc) lymphocytes and natural killer (NK) cells are key components of the host immune system protecting against tumor development and intracellular pathogens, in particular viruses. In vitro, they kill virus-infected or transformed cells by a mechanism known as ‘programmed cell death’, apoptosis [1], [2]. Tc cells employ two major mechanisms to induce apoptosis of target cells: one is via the engagement of a death receptor on the cell surface of target cells by a specific ligand (death ligand) expressed by Tc cells (i.e. CD95 (Fas)/CD95L (FasL)) [3], [4] and the other is the granule exocytosis pathway [4], [5].

The granule exocytosis pathway consists of the intracellular mobilization and release of the content of specialized organelles (cytotoxic granules) via the immunological synapse [6]. The principal granule components with cytotoxic potential are the pore forming protein perforin (perf) [7] and a family of serine-proteases named granzymes (gzm), of which gzmA [8], [9] and gzmB [10], [11] are the most abundant and best characterized. The availability of mice deficient in one or more of these cytolytic effector molecules has provided evidence of their role in controlling virus infections [12]–[17] and protection from tumours [18]–[22]. This control is facilitated by the induction of apoptosis via one of two major pathways, either the extrinsic (activated by death receptor engagement) or the intrinsic or mitochondrial-mediated (activated by cellular stress) apoptotic pathway. At least three main interconnected cellular components have been shown to be responsible for activation and/or regulation of those pathways: the caspases [23], the transcription factor p53 [24] and the Bcl-2 family, consisting of pro-apoptotic (Bid, Bak, Bax, Bim, etc.) and anti-apoptotic (Bcl-2, Bcl-X_L_, Mcl-1, etc.) proteins [25].

Viruses have been shown to interfere with the above-mentioned apoptotic mechanisms in order to optimize their replication in the host cell [26]–[28]. Ortho-poxviruses encode a number of apoptosis inhibitors. Members of this family, vaccinia virus (VACV), cowpox (CPXV) and rabbit pox (RPXV) are able to block the extrinsic, death receptor-mediated, as well as the intrinsic pathways. Extrinsic and mitochondrial apoptotic processes are inhibited through either the viral CrmA-like caspase-8/-10 inhibitor SPI-2 [29]–[31], and/or the Bak and BH3-only inhibitor F1L [32]–[34] or the Bcl-2 homologue N1L [35], respectively. Ectromelia virus (ECTV) is the causative agent of mousepox and also an orthopoxvirus [36]. Unlike VACV, CPXV and RPXV, ECTV is a natural pathogen of mice. ECTV infection of mice is therefore uniquely able to provide physiologically relevant insights into co-evolved host-pathogen relationships. Like other orthopoxviruses, ECTV is able to inhibit death receptor-induced apoptosis by SPI-2 expression [37]–[39] and UV-induced apoptosis by the ring finger protein p28 in vitro [40]. In addition, N1L and F1L orthologues are found in the ECTV genome [41].

Perf, gzmA and gzmB are all important for *in vivo* ECTV control, [13], [14]. GzmA and/or gzmB are crucial for perf-mediated induction of apoptosis by *ex-vivo* virus-immune Tc cells [42]–[45], albeit not for cellular lysis, as observed by ^51^Cr release [42], [45]. These results, together with the inability of ECTV to inhibit cellular lysis induced by perf/gzms [37], suggest that apoptotic cell death induction by gzmA and/or gzmB may be crucial for the recovery of mice from ECTV infection.

GzmB of virus-immune *ex vivo* Tc cells activates several distinct pro-apoptotic pathways including those mediated by caspases and the Bcl-2 family [43], [44]. Since gzmB is important in the control of ECTV infection and ECTV is able to inhibit a number of apoptotic pathways, we speculated that the pleiotropic effect of mouse gzmB has evolved to counteract virus evasion strategies of apoptosis. We tested this hypothesis, by using *ex vivo* ECTV-immune Tc cells to analyse the effect of ECTV infection during apoptosis induced by perf/gzmB. Our results indicate that ECTV is not able to inhibit apoptosis induced by *ex vivo* Tc cells, which is critically dependent on gzmB expression. In addition, this process correlates with reduced ECTV replication in infected target cells. Further analysis of those processes in target cells deficient in the mitochondrial or caspase apoptotic pathways reveal the relative importance of these pathways to induce apoptosis in ECTV infected cells and to inhibit ECTV replication.

## Results

### Infection of MEF cells by ECTV

To analyze the putative inhibitory effect of ECTV on apoptosis induced by gzmB we used ECTV-immune *ex vivo*-derived Tc cells from gzmA^−/−^ mice (gzmB^+^ Tc cells) as effector cells, and three mouse embryonic fibroblast (MEF) lines, wild type (wt), and two lines derived from mutant mice deficient in either the proapototic molecules Bak and Bax (MEF.BakxBax^−/−^) or the caspases 3 and 7 (MEF.Casp3x7^−/−^) as target cells. All cell lines have previously been shown to be susceptible to gzmB^+^ Tc-induced cell death [44].

We initially characterized permissibility of MEFs to infection with virulent ECTV Moscow (Mo) strain and attenuated ECTV Hampstead Egg (HE) strain. Both virus strains replicated with similar efficiency in all cell lines, as evidenced by viral plaque assays (data not shown). In addition, intracellular FACS staining of virus with anti-ECTV rabbit serum indicated that at 4 or 24 h after virus infection, similar percentages of cells became antibody positive with either Mo- or HE-ECTV strains (Fig. S1A). Furthermore, we found that mRNA of the EVM025 protein (the ECTV orthologue of the VACV F1L protein, a specific inhibitor of Bak, a proapoptotic member of the Bcl-2 family [32]–[34]) was already expressed after 4 h in Mo- or HE-ECTV infected (MOI 3∶1 (3 pfu/cell)) but not mock treated cells and also contained the BH3-like domain responsible for Bak inhibition (Fig. S1B). In addition, as reported for VV [32]–[34], Mo- and HE-ECTV inhibited apoptosis induced by the bacterial metabolite staurosporin, most probably via VACV F1L (STP. Fig. S1C). At 4–8 h after infection, cells are still viable and cell death markers are not yet detectable (Fig S1D). These data indicate that MEFs are efficiently infected by Mo- and HE-ECTV. In addition, they show that Mo/HE-ECTV express putative apoptosis inhibitors in infected targets early in the virus replication phase, before the infected cells die as a consequence of viral cytopathic effects (>24 h post infection).

### *In vivo* generation of ECTV-immune Tc cells

To generate Tc cells immune to ECTV, we infected wt or gzmA×B cluster^−/−^ mice (which are deficient in gzmA and gzmB, but also display a reduced expression of several gzmB-downstream gzm [46]) with 10^5^ PFU HE-ECTV intravenously (iv). We used the attenuated HE-ECTV, because Mo-ECTV causes severe liver and spleen destruction in gzmA×B^−/−^ mice, rendering an analysis of splenocytes not feasible [14]. At 6 d post-infection splenic CD8+ cells were enriched and found to express gzmA, gzmB and gzmK mRNA but not gzmC in wt animals (Fig. S2A). As expected [44], gzmB^+^ Tc cells expressed only gzmB and gzmK, and gzmA×B^−/−^ Tc cells only gzmK. Primary *in vitro* alloreactive B6 wt Tc cells expressed also gzmC. Proteins for gzmA and gzmB were similarly expressed in wt Tc cells, whereas, as expected, only gzmB was produced in gzmB^+^ Tc cells and neither of them in gzmA×B^−/−^ Tc cells (Fig. S2B). CFSE labelled, *ex vivo* Tc cells were tested for their cytolytic potential against mock-treated, ECTV-infected or peptide-pulsed MEFs and CFSE negative cells (target cells) were analysed for induction of the following proapoptotic features: PS translocation using Annexin V staining and membrane integrity by 7-amino actinomycin D staining (i.e. annexinV^+^/AAD^−^ equates with early apoptosis, AnnexinV^+^/AAD^+^ is indicative of secondary necrosis). We found that B6 and gzmA^−/−^ *ex vivo* Tc cells specifically induced apoptosis in ECTV-infected and peptide-pulsed MEFs (Fig. S2C). In contrast, gzmA×B^−/−^ derived Tc cells did not induce specific apoptosis (Fig. S2C). This indicates that ECTV-immune *ex vivo* Tc cells require gzmB to efficiently induce cell death markers (apoptosis: annexin V or necrosis: AAD)) in MEFs. Similar conclusions have been reached previously using peptide-pulsed targets in the LCMV mouse model [43], [44].

**Figure 1:**
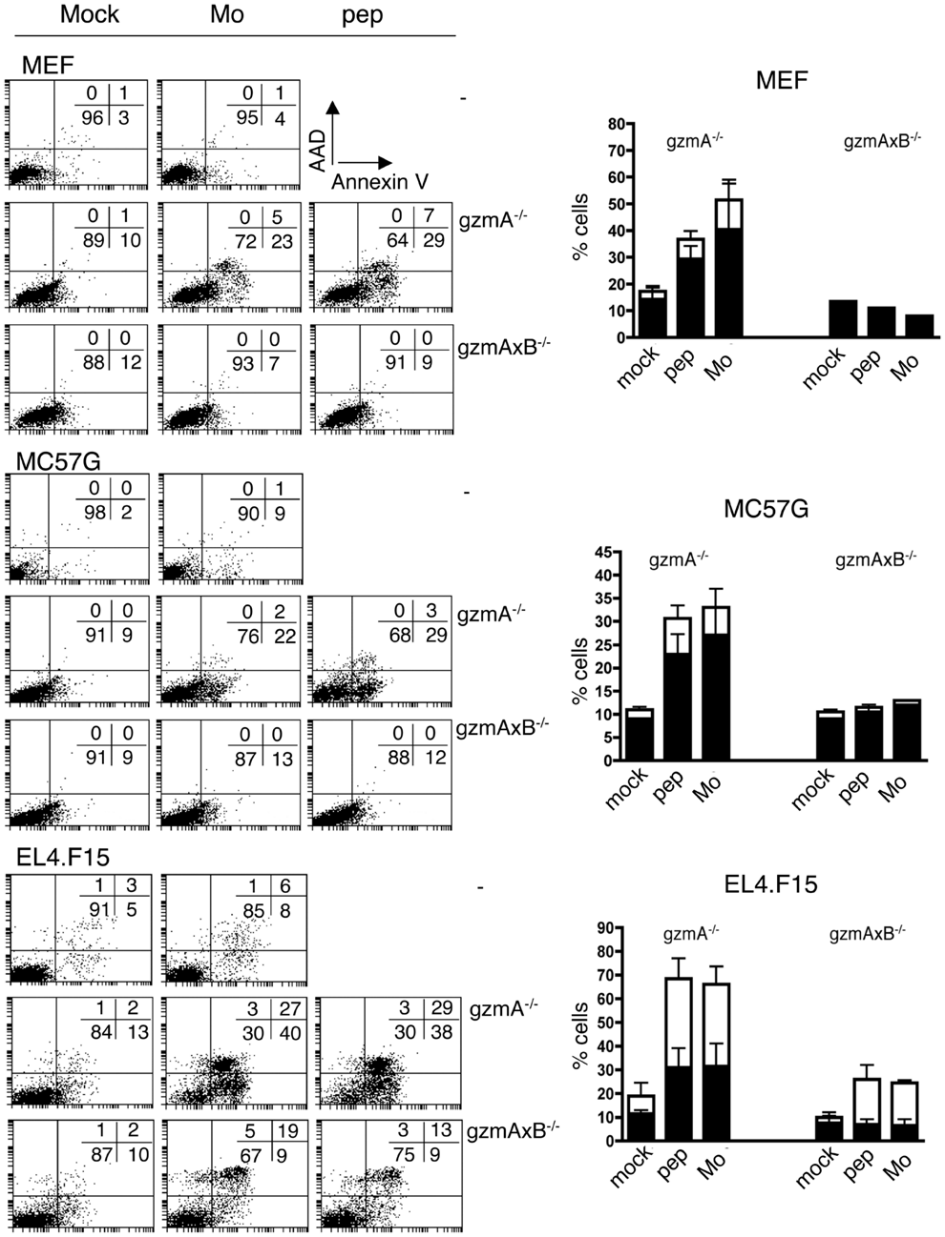
Apoptosis induced by *ex vivo* ECTV-immune Tc cells on target cells. CFSE labelled ect *ex vivo* Tc cells (C, B6, gzmA^−/−^ or gzmA×B^−/−^; D, gzmA^−/−^ or gzmA×B^−/−^) were incubated with mock-treated, peptide-pulsed or Mo-ECTV-infected (5 h, MOI = 3) MEF.wt, EL4 and MC57G cells for 3 h. Early (AV^+^/AAD^−^, filled bars) and late apoptotic/necrotic (AV^+^/AAD^+^, empty bars) cells in the CFSE^−^ negative cell population (target cells) were analysed by FACS. Data are given as mean+/−SEM of 2 independent experiments.

Results obtained with different target cells, MC57G or EL4.F15 cells, confirmed the results obtained with MEFs (Fig. 1 vs Fig. S2C): Only gzmB^+^ Tc cells, but not gzmA×B^−/−^ derived ECTV-immune *ex vivo* Tc cells could induce significant apoptosis on peptide-pulsed or ECTV-infected target cells. These results are consistent with the high susceptibility of gzmA×B^−/−^ mice to ECTV infection and suggest that pro-apoptotic processes induced by perf and gzms may be responsible for the gzm-dependent recovery from ECTV infection. In addition, they show that lack of gzmA does not affect the pro-apoptotic potential of ECTV-immune *ex vivo* Tc cells in vitro. For these reasons, we used ECTV-immune *ex vivo* Tc cells from gzmA^−/−^ mice (gzmB^+^ Tc cells) as effector cells in all further experiments.

### ECTV does not inhibit PS translocation induced by *ex vivo* immune gzmB^+^Tc cells

**Figure 2:**
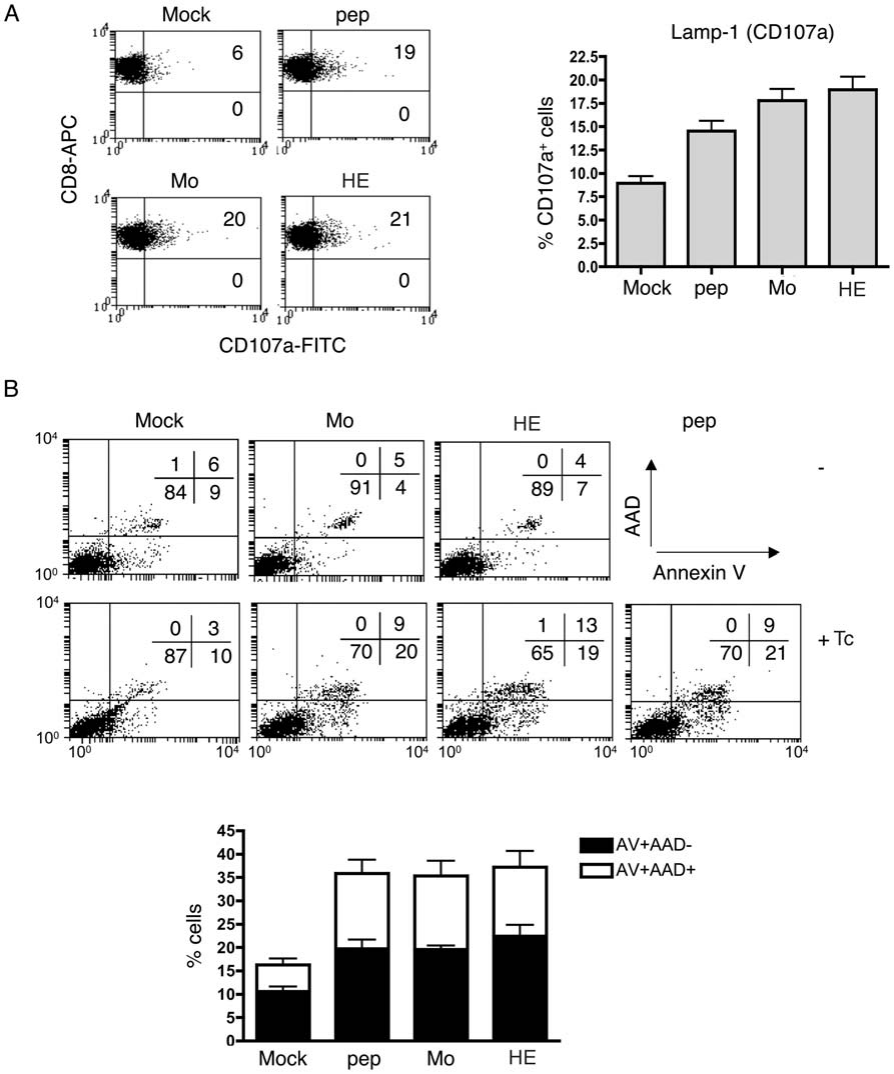
Degranulation of *ex vivo* gzmB^+^ Tc cell and PS translocation on target cells. MEF.wt cells were mocked-treated, peptide-pulsed or infected (5 h) with Mo- or HE-ECTV. A, MEF-wt target cells were incubated for 5 h with *ex vivo* Tc cells in the presence of monensin and anti-CD107a (FITC), followed by FACS staining with anti-CD8 (APC) and anti-CD107a (FITC). Representative dot plots (with % of cells in top right and bottom right quadrants) and means+/−SEM of percent CD107a^+^ CD8^+^ cells are shown from 5 independent experiments performed by duplicate. B, MEF-wt target cells were incubated with CFSE labelled *ex vivo* Tc cells for 3 h and PS translocation (annexin V-PE staining) and membrane integrity (AAD staining) were analysed by FACS in the CFSE^−^ negative cell population (target cells). Data are given as mean+/−SEM of 10 independent experiments.

As we wanted to see whether ECTV virulence correlated with inhibition of gzmB-induced apoptosis, we compared apoptosis induction by ex vivo gzmB^+^ Tc cells on MEFs infected with either the virulent Mo-ECTV or the attenuated HE- strain. As controls we used peptide-pulsed or mock-treated MEFs. We first determined whether Mo- and HE-ECTV, render target cells similar recognizable to ECTV-immune *ex vivo* Tc cells. We analysed Tc cell degranulation (by measuring the number of Tc cells expressing the lysosomal marker, CD107a (Lamp1), on their cell membrane, [44]) after incubation with ECTV-infected, peptide-pulsed or mock treated MEFs (Fig 2A). No significant differences in the proportion of CD107a^+^ Tc cells after incubation with MEF infected with either strain of ECTV was found. Thus, *ex vivo* ECTV-immune Tc cells recognise MEF with similar efficiency after infection with either virulent or attenuated ECTV, and degranulation correlated with apoptosis induced by gzmB^+^ Tc cells on MEFs (Fig 2B). GzmB^+^ Tc cells induced apoptosis to the same extend on peptide-pulsed and Mo- or HE-ECTV infected MEFs (4–5 h post-infection) compared to mock-treated targets (Fig. 2B). Together with the previous findings that apoptosis (measured by PS translocation) was neither affected by virulent Mo-ECTV in two other cell lines tested, EL4 and MC57G (Fig. 1), the data suggest that cell death in these target cells is not altered by the virus.

Together, these findings indicate that gzmB of *ex vivo* ECTV-immune Tc cells is able to overcome the anti-apoptotic potential of ECTV that is evident against staurosporine.

### ECTV does not inhibit other apoptotic markers induced by *ex vivo* gzmB^+^ Tc cells

**Figure 3:**
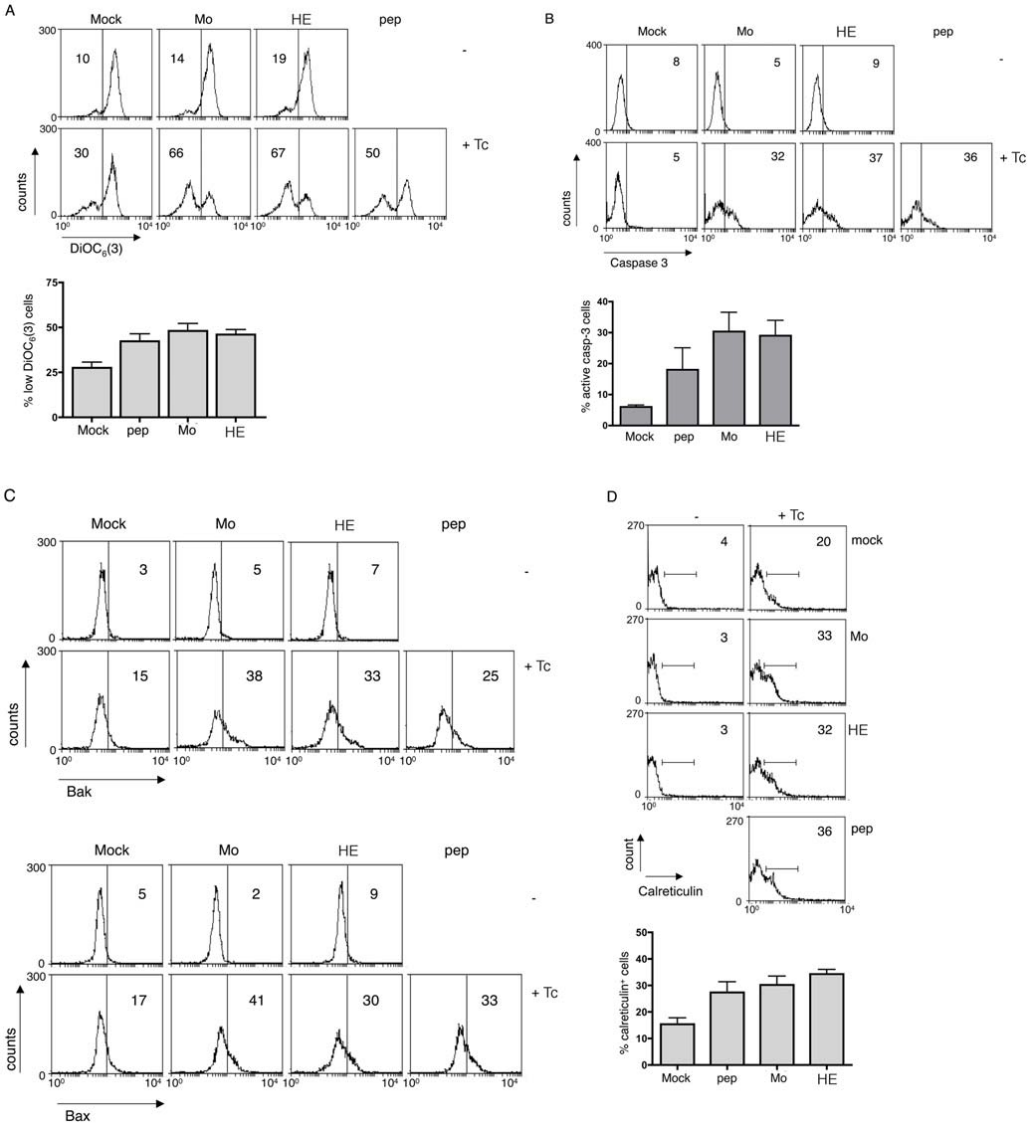
*Ex vivo* gzmB^+^ Tc cell-induced mitochondrial damage, caspase 3 activation, Bak/Bax conformational change and calreticulin translocation in ECTV-infected targets. Mock-treated, peptide-pulsed or Mo-ECTV-infected MEF.wt cells were incubated with CFSE labelled (C) or unlabelled (A, B) *ex vivo* Tc cells for 3 h and mitochondrial membrane potential (DiOC_6_(3), A) caspase 3 activation (anti-active caspase 3-FITC antibody, B) were analysed in the CD8^−^ population by FACS or Bak and Bax conformational change (anti-N-terminal Bak/Bax antibodies, C) and calreticulin translocation (D) in the CFSE^−^ cell population. Data are given as mean+/−SEM of percent positive cells from 4 (A), 3 (B), 2 (C) or 4 (D) independent experiments.

Although gzmB^+^ Tc cell-mediated PS translocation was not inhibited in MEFs infected with ECTV viruses, we investigated whether other pro-apoptotic features, elicited by gzmB, may be affected. ECTV-immune *ex vivo* gzmB^+^ Tc cells were incubated with MEFs either mock, peptide-pulsed or Mo- or HE-ECTV-infected and monitored for a) loss of mitochondrial membrane potential by staining with DiOC_6_(3), (Fig. 3A), b) caspase 3 activation by staining with anti-active caspase-3 antibody, (Fig. 3B) and c) conformational changes of Bak and Bax by staining with mAbs that recognise N-terminal epitopes of Bak and Bax, respectively, which are exposed only upon their functional activation (Fig. 3C). None of the parameters tested were significantly affected as a result of ECTV infection (Fig. S1D), although an increased caspase 3 activation in ECTV infected target cells was observed (Fig. 3B), which possibly reflected early caspase 3 induction by ECTV itself [47].

### *Ex vivo* ECTV-immune gzmB^+^ Tc cells induce calreticulin translocation, independently of ECTV infection

It was possible that ECTV affects the quality of cell death thereby evading specific immunity in order to hide of the immune system. It is well established that, under certain conditions, dying cells express particular signals relevant for the induction of immune responses, including immunological memory formation. One of these signals is calreticulin, which may be exposed on membranes of dying cells [48], [49]. We tested calreticulin exposure on the outer cell membrane of Tc cell-treated, ECTV-infected target cells. *Ex vivo* gzmB^+^ Tc cells were incubated with MEFs (mock, peptide-pulsed or Mo- or HE-ECTV-infected) and calreticulin exposure was analysed by FACS (Fig. 3D). There was no difference in calreticulin membrane expression induced irrespective whether MEFs were peptide-pulsed or infected with ECTV.

### ECTV inhibits apoptosis induced by ex vivo ECTV- immune gzmB^+^ Tc cells in target cells lacking Bak and Bax

**Figure 4:**
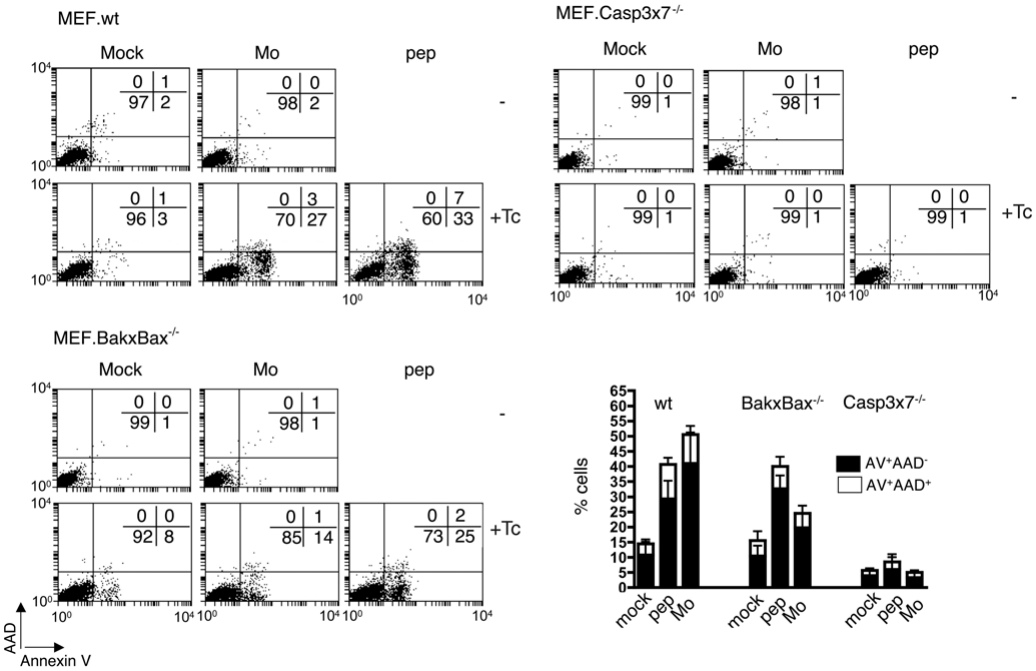
*Ex vivo* gzmB^+^ Tc cell-induced PS translocation in target cells lacking casp 3 and casp 7 or Bak and Bax. Mock-treated, peptide-pulsed, or Mo- or HE-ECTV-infected MEF.wt, Casp3x7^−/−^ or BakxBax^−/−^ cells were incubated with CFSE labelled *ex vivo* Tc cells for 3 h. Percent early (AV^+^/AAD^−^, filled bars) and late apoptotic/necrotic (AV^+^/AAD^+^, empty bars) cells in the CFSE^−^ negative cell population (target cells) are given as mean+/−SEM of 4 independent experiments.

CPXV SPI-2 (CrmA) [50], but not ECTV SPI-2 [39] was shown to inhibit gzmB in a cell free system or after transient induced expression in a cell line, respectively. Our data indicate that ECTV encoded inhibitors are not able to generally block gzmB^+^Tc cell-mediated apoptosis in wt-MEFs. Thus the question arises; if gzmB's broadly induced apoptosis can only be mediated by one of the two major pathways in virus infected target cells. We therefore used MEF.BakxBax^−/−^ and MEF.Casp3x7^−/−^ (mock-treated, peptide-pulsed or Mo-ECTV-infected) target cells with *ex vivo* ECTV-immune gzmB^+^ Tc cells and analysed the induction of pro-apoptotic features (Fig. 4). In agreement with the LCMV model [44], we found that PS translocation induced by ECTV-immune *ex vivo* gzmB^+^CTLs on both MEF.wt targets, peptide-pulsed and Mo-ECTV-infected, was completely dependent on caspases-3 and/or -7.

This was paralleled in the ECTV system for peptide-pulsed MEF.BakxBax^−/−^, which were as susceptible to gzmB^+^ Tc cell-induced PS exposure as peptide-pulsed MEF.wt. In contrast Mo-ECTV-infected MEF.BakxBax^−/−^ were around 50% less susceptible than Mo-ECTV-infected MEF.wt. This suggests that, in the absence of Bak and/or Bax, ECTV interferes with gzmB*^+^* Tc cell-disrupted plasma membrane asymmetry.

Since gzmB-facilitated PS translocation is critically dependent on casp3x7 [44], but amplified via mitochondrial processes, including apoptosome/caspase-9 complex formation, this finding provides evidence that the mitochondrial pathway is critical for efficient gzmB-induced apoptosis of ECTV-infected cells.

### GzmB^+^ but not gzmA×B^−/−^ Tc cells inhibit ECTV replication *in vitro* by a caspase dependent mechanism

**Figure 5:**
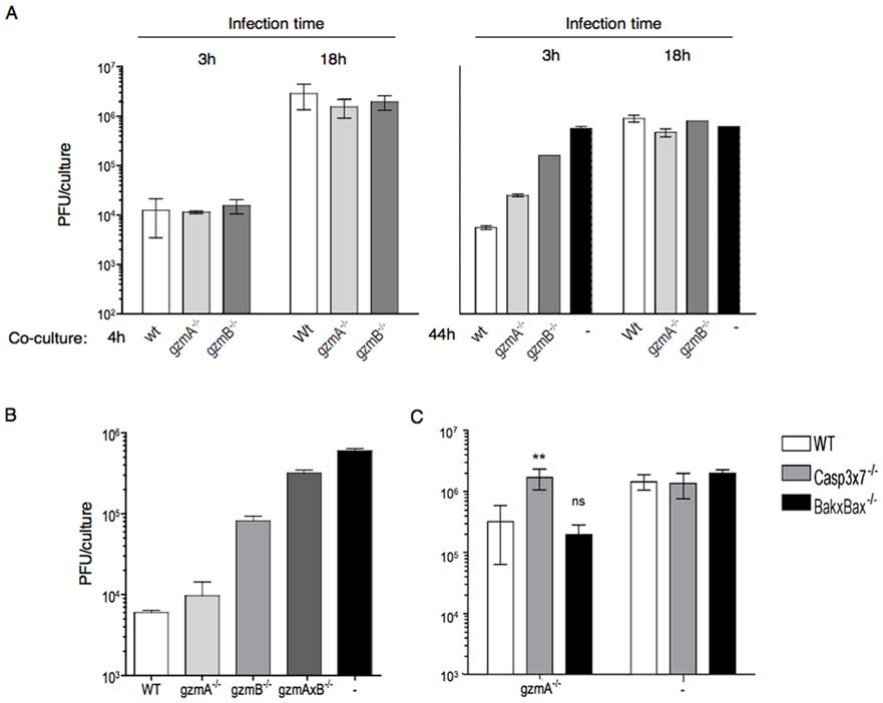
*Ex vivo* Tc cells need gzmB to inhibit ECTV replication in vitro by a casp3x7 dependent process. A, B, MEF.wt cells were infected for 3 or 18 h before being subjected to attack by *ex vivo* Tc cells from ECTV-immune WT (empty bars), gzmA−/− (light bars), gzmB−/− (dark bars) or gzmA×B−/− (filled bars) mice for 4 (A, left panel) or 44 h (A, right panel, B). Cell suspensions were removed and analysed for viral titers by plaque assay. C, MEF.wt, Casp3x7^−/−^ or BakxBax^−/−^ cells were infected for 3 h before being subjected to attack by *ex vivo* Tc cells from ECTV-immune gzmA^−/−^ mice for 44 h. Cell suspensions were removed and analysed for viral titers by plaque assay. Data in A and B are from separate experiments. Data are given as mean+/−SD from two to four replicate cultures. Similar results were obtained in a separate experiment. ** statistically significant (n = 3–4, P = 0,007); ns, no statistically significant (n = 3–4, P>0,05). Analysed by a two-tailed unrestricted t-student test comparing casp3x7 or BakxBax with wt.

To address the question, whether differential inhibition of gzmB-induced apoptosis by ECTV affects virus replication, we infected MEF target cells with Mo-ECTV for 3 or 18 h before subjecting them to attack by *ex vivo* wt B6, gzmB^+^ or gzmA^+^ (gzmB^−/−^) Tc cells. Viral titers in cultures were analysed after 4 or 44 h of co-incubation. In those targets that had been infected for 18 h before exposure to virus-immune Tc cells, high titers were observed already at the beginning of the co-cultures (Fig. 5A). These titers did not change over the subsequent 44 h, independent on the presence or absence of virus-immune Tc cells. This suggests that virus had completed a replication cycle before exposure to virus-immune Tc cells, and that virtually all susceptible target cells had been infected and maximally replicated virus. However, early (3 h) after target infection, low titers were observed, consistent with the eclipse phase of virus infection (Fig 5A). Spleens of the HE-ECTV infected mice that provided the virus-immune Tc cells had no detectable virus at the time of sacrifice (not shown), ruling out a contamination from this source. WT Tc cells were then able to completely suppress any viral replication in these targets, since viral titers in their cultures did not increase over the next 44 h, whereas in the absence of Tc cells, detectable virus increased 100-fold during this time (Fig 5A). In contrast, exposure to gzmA^+^ (gzmB^−/−^) and gzmB^+^ (gzmA^−/−^) virus-immune Tc cells resulted in a graded suppression of viral replication.

Next, we tested the ability of gzmA^+^, gzmB^+^ or gzmA×B^−/−^ Tc cells to suppress viral titers in targets infected shortly before Tc cell attack (Fig 5B). We found an increasing ability of Tc cells to suppress ECTV replication, depending on whether they expressed gzmA, gzmB or both. This viral suppression activity *in vitro* correlated with apoptosis induced by those Tc cells (Fig. 1 and Fig. S2C) and indicated that gzmB's ability to overcome ECTV inhibition of apoptosis likely has direct effects on virus replication and/or clearance. Furthermore, gzmA also clearly showed an antiviral effect in these assays, despite not contributing to any of the analyzed apoptosis parameters (Fig. S2C). Finally, we tested whether the increased resistance of ECTV-infected MEF.BakxBax^−/−^ and MEF.Casp3x7^−/−^ cells to apoptosis induced by gzmB^+^ Tc cells yielded a higher ECTV replication rate in those cells. As shown in the experiment in Fig 5C we found that upon incubation with gzmB^+^ Tc cells, ECTV replication was around 10× higher in MEF.Casp3x7^−/−^ compared to both, MEF.wt or BakxBax^−/−^ cells. An independent experiment yielded similar results with a 100-fold difference between wt and casp3x7^−/−^ cells (not shown). This indicated that partial inhibition of gzmB^+^Tc cell mediated apoptosis by ECTV in Bak and Bax-deficient target cells does not interfere with suppression of virus replication in host cells. In addition, the data show for the first time that casp3x7 are not only important during Tc-mediated and gzmB-facilitated apoptosis, but critically involved in the control of ECTV replication *in vitro* and indicate the biological relevance for the caspase dependent apoptotic pathway in gzmB-mediated control of ECTV.

## Discussion

Tc cells [51], and gzmA/B [52] are essential for the recovery of mice from primary infection by ECTV, mousepox, and it has been assumed that the two gzms mediate their protective effect via apoptosis induction of infected cells through the granule exocytosis pathway. It has previously been shown that ECTV resistant B6 mice become 10 times more susceptible when gzmA is absent and 10^7^ times more susceptible when both gzmA and B are lacking, as demonstrated in the gzmA×B^−/−^ cluster knock-out mice [14]. Most probable, the latter finding is due to the inability of gzmA×B^−/−^ Tc cells to induce pro-apoptotic processes, such as PS translocation or membrane permeabilization in target cells, as demonstrated here with three different types of target cells (EL4 lymphoma, embryonic fibroblasts and MC57G fibrosarcoma). This contrasts with Tc cells from gzmA^−/−^ mice (gzmB^+^ Tc cells), which exhibit no defect in their ability to induce apoptosis in ECTV-infected targets. These results are in agreement with previous findings in the LCMV-system [42]–[45]. Moreover, it would be expected that if ECTV was able to efficiently block gzmB-dependent *in vivo* processes, including apoptosis, gzmA^−/−^ mice should be as susceptible to ECTV infection as gzmA×B^−/−^, which is not the case [14], [53]. This is also in agreement with reports showing that gzmB is not inhibited by ECTV encoded serpins (including SPI-2/CrmA) [38], [39]. Supporting these arguments are the data presented here that apoptosis induced by gzmB^+^ Tc cells is not abolished by ECTV infection. Although, gzmC and gzmF are down regulated in the gzmA×B−/− cluster KO mouse, the participation of those gzms in ECTV resistance is unlikely because they are not expressed at detectable levels in ECTV-immune Tc cells from B6wt and gzmA−/− mice.

The importance of gzmA and gzmB for ECTV control is further supported by our present finding that *ex vivo* ECTV-immune Tc cells from gzmA×B^−/−^ mice are unable to inhibit ECTV replication *in vitro* in infected target cells in spite of the presence of functional perforin present in those effectors. In contrast Tc cells from wt or gzmA−/− (expressing gzmB) mice reduced ECTV titres more than 100-fold, which correlated with their ability to induce apoptosis in those infected target cells. This was expected since the virus needs a host cell to replicate and ensure that viral progeny is disseminated to infect other cells. However, the timing of the Tc cell encounter with the infected cell appears to be critical. At late stages of infection, apoptosis induction of infected cells appears to have no beneficial effect because viral progeny formation is already complete, and lysis may in fact facilitate the liberation of viral particles. Consistent with this idea, we found that wt or gzmB*^+^*Tc cells are not able to reduce viral titers if virus infection had progressed for 18 h (in contrast to infection for 3 h) prior to Tc cell addition. Interestingly, gzmA also showed an inhibitory effect on viral replication *in vitro*, consistent with its demonstrable role in resistance of mice from mousepox. The fact that we did not detect any contribution of gzmA to target cell apoptosis means that either it mediates apoptosis by pathways we did not analyze, or, more likely, given its lack of cytotoxicity under some circumstances [55], that its antiviral function is mediated by pathways other than apoptosis induction.

It is known that immune-mediated apoptosis induction, as the major threat to ECTV replication success, is mediated via several proapoptotic pathways [43], [44]. It is therefore likely that the full range of cell death pathways induced by gzmB has evolved due to selective pressure from pathogens like ECTV via its strategy to inhibit selective apoptosis pathways. In this respect, one could speculate that, in the course of its future co-evolution, ECTV may acquire more diverse apoptosis inhibiting capabilities. However, unimpaired virus replication may not necessarily constitute a successful viral strategy as an early demise of the host would curtail wide virus dissemination. On the other hand, it is possible that ECTV at least partially inhibits gzmB-mediated apoptosis induction. The comparison of peptide-pulsed target cells, which are labelled at 100% but targeted only by a minority of anti-ECTV Tc cells on the one hand, and virus-infected target cells, which are only infected at about 80% but display Tc cell determinants for the whole spectrum of anti-ECTV Tc cells on the other hand, would make it difficult to detect such a partial inhibition.

We also did not find any viral interference with calreticulin membrane translocation after Tc cell attack. This is important since it is now evident that dying cells signal the immune system to get activated against antigens contained in those cells and one of most critical is the calreticulin translocation. This indicates that ECTV does not affect its immunogenicity, i.e. proper phagocytosis of infected target cells and subsequent promotion of anti-viral/protective Tc responses [48], [49].

Using MEF.BakxBax^−/−^ cells and LCMV-immune *ex vivo* gzmB^+^Tc cells, we have previously shown that Bak and Bax were dispensable for PS translocation and caspase 3 activation induced by gzmB [44]. However, Bak and Bax were required for gzmB^+^Tc cell-induced cytochrome c release from mitochondria, a process that may contribute to caspase-3 activation via an amplification loop, including the apoptosome/caspase-9 complex [56]. We tested if ECTV infection of target cells interferes with the processes established in the LCMV system by using ECTV-immune *ex vivo* gzmB^+^ Tc cells on ECTV –infected MEF.BakxBax^−/−^ or MEF.Casp3x7^−/−^ targets. Although ECTV did not affect apoptosis induction by gzmB^+^Tc cells on MEF.wt, it partially inhibited apoptosis in MEF.BakxBax^−/−^ cells. These data highlight the importance of the mitochondrial apoptotic pathway, triggered by gzmB, for efficient elimination of ECTV-infected cells. However ECTV replication was inhibited as much in MEF.BakxBax−/− cells as in wt cells, but not at all when casp3x7 were absent. This result indicates that casp3x7 are not only critically involved in apoptosis induced by mouse gzmB, but also in the control of ECTV replication *in vitro*. This finding emphasizes the importance of effectors caspases during apoptosis induced by gzmB and at the same time it suggests that mitochondrial amplification of gzmB-induced apoptosis is not needed to inhibit ECTV replication.

The complexity of cell death induction by antiviral Tc cells in virus-infected cells may thus reflect the evolutionary balance achieved between pathogen and host, in which the former needs to replicate sufficiently to ensure further spread, yet not enough to kill its host prematurely.

In conclusion, the pleiotropic nature of cell death induced by gzmB appears to be crucial to overcome the ability of ECTV to interfere with apoptosis induced by Tc cells. Since ECTV is a natural mouse pathogen and gzmB appears to be important for its *in vivo* control, an understanding of their interplays is of great importance in understanding host/parasite relationships.

## Materials and Methods

### Ethics statement

All experiments involving animals were performed in accordance with Australian National University (ANU) Animal Experimentations Ethics Committee.

### Mouse strains and viruses

Inbred C57BL/6 (B6), and mouse strains deficient for gzmA (gzmA^−/−^), and gzmA×B (gzmA×B^−/−^), bred on the B6 background were maintained at the John Curtin School of Medical Research, Canberra and analysed for their genotypes as described [18]. Male mice of 8 to 10 weeks of age were used in all experiments and were performed in accordance with the local animal ethics authority.

The mouse orthopoxvirus ectromelia strains Moscow (Mo-ECTV) and Hampstead Egg (HE-ECTV) have been described [37].

### Generation of ex-vivo CD8^+^ cells

Mice were infected with 10^5^ pfu HE-ECTV i.v. according to established protocols [14]. On d6 post infection (p.i.), CD8^+^ cells were positively selected from spleen using α-CD8-MicroBeads (Miltenyi Biotec, Bergisch Gladbach, Germany) with an autoMACS (Miltenyi Biotec) and resuspended in MEM/5% FCS prior to use in cytotoxic assays. Purity of selected CD8^+^ cells was assessed by FACS staining and found to be between 95–98%.

### Cell lines, cell culture and reagents

SV40 transformed mouse embryonic fibroblasts (MEFs) were cultured in MEM supplemented with 10% FCS and 2-mercaptoethanol (10^−5^ M) at 37°C, 7% CO_2_. BaxxBak^−/−^ MEFs were generated as described previously [57] and were generously provided by the S.J. Korsmeyer laboratory. Caspase 3x7^−/−^ MEFs were generated as described previously and generously provided by Richard A. Flavell [58]. The genotype of the various MEF cells was periodically tested by genomic PCR and western blot analysis. The mouse cell lines EL4.F15 and MC57G have been previously described [42]. In some cases the general caspase inhibitor Ac-ZVAD-fmk (Bachem; 100 µM) was added to cell cultures as described [44].

### Cell peptide pulsing and in vitro virus infection

Wt, Casp3x7^−/−^, BakxBax^−/−^ MEFs, EL4 or MC57G cells were incubated with 10^−5^ M of the ectromelia immunodominant K^b^ restricted peptide TSYKFESV-OH [59] for 2 h at 37°C. Subsequently, cells were washed and used for the corresponding assays.

Wt, Casp3x7^−/−^, BakxBax^−/−^ MEFs, EL4 or MC57G cells were infected with Mo or HE-ECTV at a multiplicity of infection of 3 pfu per cell (MOI 3∶1) for 4–5 h at 35°C. Subsequently, cells were washed and used for the corresponding assays.

### RT-PCR

Total RNA was extracted from up to 5×10^6^ cells, using the QIAshredder spin columns, the RNeasy Mini Kit and the RNase-free DNase Kit (all from Qiagen, Hilden, Germany) according to manufacturer's instructions and converted to cDNA as described [60]. Specific transcripts were amplified by using specific primers. The sense/antisense primers for *gzmA*, gzmB, gzmC, gzmK and *GADPH* and the PCR conditions have been previously described [44].

### EVM025 (F1L) sequencing

Total RNA was isolated from MEF.wt cells previously infected with Mo or HE-ECTV (MOI 3∶1, 4 h, 35°C) using the QIAshredder spin columns, the RNeasy Mini Kit and the RNase-free DNase Kit (all from Qiagen, Hilden, Germany) according to manufacturer's instructions and converted to cDNA as described [60]. Full EVM025 cDNA was amplified by using the primers: 5′-ACGGGATCCATGGACAATAGTATTTTGTCG-3 (forward) and 5′-TCGGGATCCTCATATCATGTATTTGAGAGTC-3 (reverse). PCR was done as follows: 95°C/4 min. (1×); 95°C/30 sec., 51°C/1 min., 72°C/2 min. (5×); 95°C/30 sec., 58°C/1 min., 72°C/2 min. (35×); 72°C/7 min. PCR products were separated in agarose gel electrophoresis and fragments were purified and sequenced by using the BigDye Terminator v3.1 Cycle Sequencing Kit and a capillary-based Applied Biosystems 3730 DNA Analyzer at the John Curtin School of Medical Research's DNA sequencing facility.

### Flow cytometry

Cell populations were analysed for cell surface marker expression and/or intracellular expression of gzmA and gzmB by FACS as described [44]. Rabbit immune serum (IS) specific for mouse gzmA (αmgzmA) and mouse gzmB (α-mgzmB) has been described [61].

### Lamp-1 mobilization assay

Lamp-1 (CD107a) expression in the cell membrane of *ex vivo* Tc cells was analysed as described [44]. Mock-treated, Peptide-pulsed or ECTV infected MEF cells were incubated with *ex vivo*-derived ECTV-immune Tc cells at 2∶1 effector∶target cell ratio (triplicates, 4×10^4^ targets and 8×10^4^ Tc cells/well). Subsequently, the mixed cell population was treated with 1 µl (final dilution 1∶200) of α-Lamp-1-FITC antibody (BD) in the presence of monensin for 6 h at 37°C, 7% CO_2_. Non-attached cells were collected, labelled with α-CD8-APC antibody and analysed by FACS.

### Analysis of pro-apoptotic processes

To analyse cell death induced by *ex vivo* Tc cells in target cells, mock-treated, peptide-pulsed or ECTV-infected cells were incubated with *ex vivo*-derived ECTV-immune Tc cells at 10∶1 effector∶target cell ratio for 3 h at 37°C, 7%CO_2_ [43]. In some cases, Tc cells were previously labelled with CFSE 1 µM. Subsequently, different apoptotic parameters were tested in the target population (CD8^−^ or CFSE^−^) by FACS with a FACScan (BD) and CellQuest® software as follows:

### a Cell membrane and mitochondrial membrane perturbations

PS exposure and 7-amino-actinomycin D (7-AAD) uptake was analysed by FACS using annexin V-PE and 7-AAD from BD Pharmingen according to manufacturer's indications. The mitochondrial membrane potential was measured with the fluorescent probe 3,3′-dihexyloxacarbocyanine iodide (DiOC_6_(3), Molecular Probes) [44].

### b Caspase-3 activation

Caspase 3 activation was analysed by FACS as described [44]. Cells were fixed with 4% paraformaldehyde (PFA) and incubated with a FITC labelled monoclonal antibody against the active form of caspase-3 (clone C92605, BD Pharmingen) diluted in 0.1% saponin in PBS. After two washes with 0.1% saponin in PBS, cells were resuspended in 1% PFA/PBS and analysed by FACS.

### c Conformational change of Bax and Bak

Bak and Bax conformational change was analysed by FACS as described [44]. Briefly, cells were fixed in 4% PFA, permeabilized with 0.1% saponin in PBS/5% FCS and incubated with polyclonal rabbit α-Bak (NT, Upstate Biotechnology), polyclonal rabbit α-Bax (NT, Upstate Biotechnology) or purified rabbit IgG (control). After washing (2×) with 0.1% saponin in PBS, cells were incubated with α-rabbit-Alexa 647 antibody (Invitrogen) in 0.1% saponin/PBS/5% FCS, washed twice in 0.1% saponin/PBS, resuspended in 1% PFA/PBS and analysed by FACS.

### d Analysis of calreticulin translocation

Calreticulin translocation to the outer plasma membrane was analysed as described [49]. Briefly, cells were incubated with rabbit anti-calreticulin pAb (Biomol) (30 min, 4°C), followed by goat anti-rabbit-alexa647 secondary antibody (30 min, 4°C). After washing twice PBS, cells were resuspended in a buffer containing 7-AAD and calreticulin was analysed in the 7-AAD^−^ cell population by FACS.

### Analysis of viral titers during Tc cell induced cell death

Anti-ECTV CD8+ Tc cells were generated and isolated as described above, except that mice were immunized with 10^4^ instead of 10^5^ pfu HE-ECTV, such that none of the infected mice contained any detectable ECTV in their spleens at the time of harvest. Effector and MEF target cells (infected for the indicated times before the assay) were co-incubated at 10∶1 effector∶target cell ratio at 37°C, 7%CO_2_. After the indicated times of co-incubation, thoroughly mixed cell suspensions were analyzed for ECTV by plaque assay.

## Supporting Information

Figure S1 Characterization of ECTV infection in MEF.wt cells. A, MEF.wt cells were mocked treated or infected with Mo- or HE-ECTV (moi 3∶1) for 5 or 18 hours. ECTV antigen was detected by using a polyclonal rabbit anti-ECTV serum and analysed by FACS as described in materials and methods. Representative histograms (left panel) and means+/−SEM of percent ECTV-antigen-positive cells from 6 (5 h) or 2 (18 h) independent experiments (right panel). B, MEF.wt cells were mocked treated or infected with Mo- or HE-ECTV (moi 3∶1) for 4 or 8 hours. Total RNA was isolated, EVM025 cDNA (F1L) was amplified, cloned and cDNA was analysed by agarose gel electrophoresis (left panel). The bands were purified and sequenced. Amino acid sequence alignments (right panel) are shown for the F1L BH3-like domain from Mo- and HE-ECTV and Vaccinia virus (Obtained from [34]). C, MEF.wt cells were mocked-treated or infected (5 h) with Mo- or HE-ECTV and subsequently treated with staurosporin 500 nM. After 12 h PS translocation (annexin V-PE staining) and membrane integrity (AAD staining) were analysed by FACS. Data are given as mean+/−SEM of 3 independent experiments. D, MEF.wt cells were mocked-treated, EV-Kb-peptide-pulsed or infected with Mo- or HE-ECTV (moi 3∶1) for 5 h. Cell were washed and incubated for 3 hours more and PS translocation (annexin V-PE staining) and membrane integrity (AAD staining), mitochondrial membrane potential (DiOC6(3)) and calreticulin expression on the cell membrane were analysed by FACS as described in materials and methods.
(1.57 MB TIF)

Figure S2 Characterization of ex vivo Tc cells from ECTV infected mice. CD8+ cells (anti-ECTV ex vivo Tc cells) were isolated by autoMACS from six-day HE-ECTV immune WT (B6), gzmA−/− or gzmA×B−/− mice. A, Total RNA was isolated and gzmA, gzmB, gzmC, and gzmK transcripts were analysed by RT-PCR. EL4 served as negative and day 6 primary allogeneic Tc cells as positive control. GADPH was analysed as housekeeping gene. B, intracellular gzmA and gzmB protein expression detected with anti-gzmA or anti-gzmB rabbit antiserum. Data shown are representative histograms of anti-gzm fluorescence (top panel) and means+/−SEM of percent gzm+ CD8+ cells (bottom panel) from 3 independent experiments.
(2.07 MB TIF)
