# Supplementary figures and images for: Caspase-Dependent Inhibition of Mousepox Replication by gzmB
Source: PLoS One. 2009 Oct 19;4(10):e7512. doi: 10.1371/journal.pone.0007512 (PMC2759507; doi:10.1371/journal.pone.0007512)

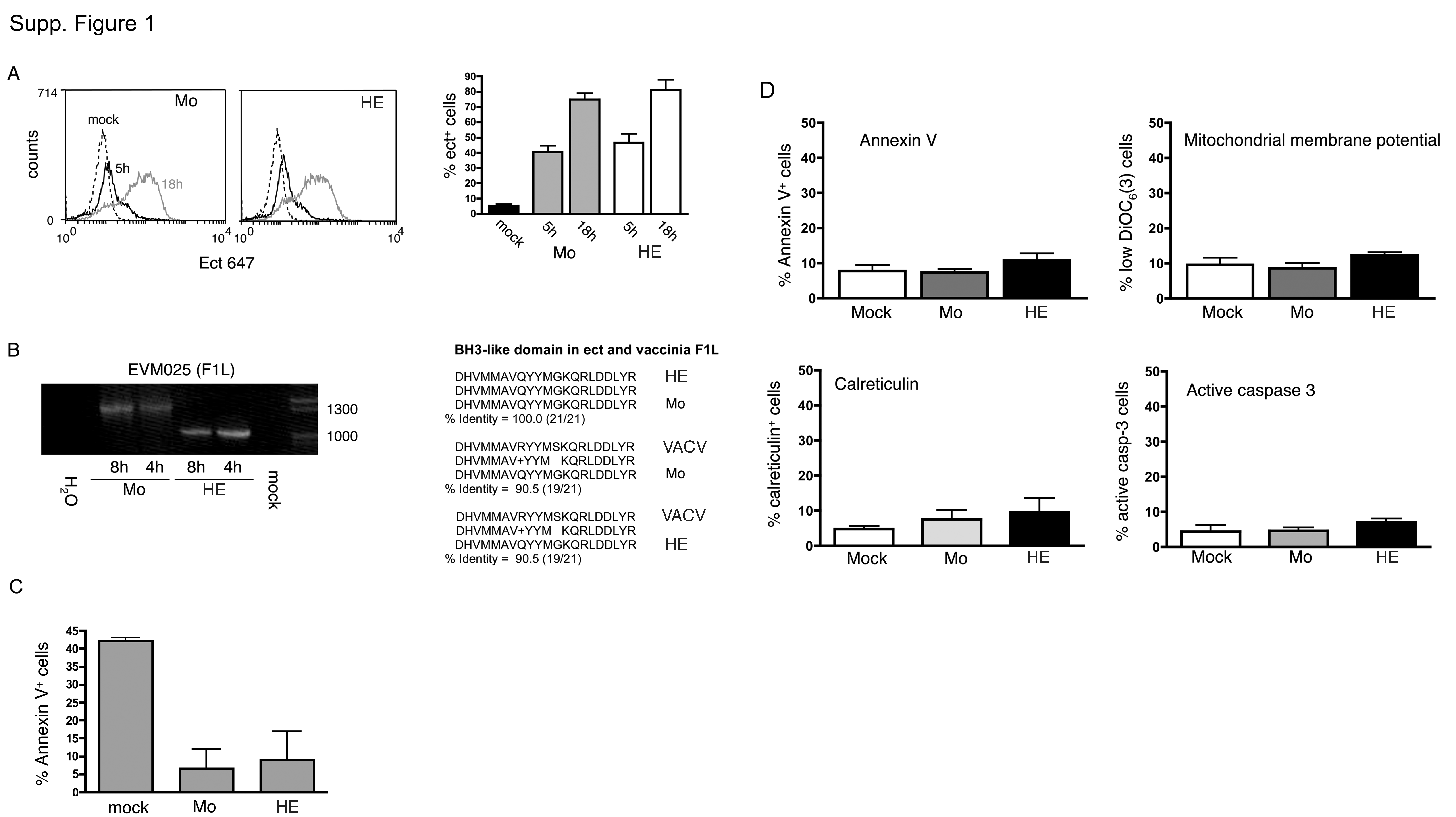

Supplement: Figure S1 — Characterization of ECTV infection in MEF.wt cells. A, MEF.wt cells were mocked treated or infected with Mo- or HE-ECTV (moi 3∶1) for 5 or 18 hours. ECTV antigen was detected by using a polyclonal rabbit anti-ECTV serum and analysed by FACS as described in materials and methods. Representative histograms (left panel) and means+/−SEM of percent ECTV-antigen-positive cells from 6 (5 h) or 2 (18 h) independent experiments (right panel). B, MEF.wt cells were mocked treated or infected with Mo- or HE-ECTV (moi 3∶1) for 4 or 8 hours. Total RNA was isolated, EVM025 cDNA (F1L) was amplified, cloned and cDNA was analysed by agarose gel electrophoresis (left panel). The bands were purified and sequenced. Amino acid sequence alignments (right panel) are shown for the F1L BH3-like domain from Mo- and HE-ECTV and Vaccinia virus (Obtained from [34]). C, MEF.wt cells were mocked-treated or infected (5 h) with Mo- or HE-ECTV and subsequently treated with staurosporin 500 nM. After 12 h PS translocation (annexin V-PE staining) and membrane integrity (AAD staining) were analysed by FACS. Data are given as mean+/−SEM of 3 independent experiments. D, MEF.wt cells were mocked-treated, EV-Kb-peptide-pulsed or infected with Mo- or HE-ECTV (moi 3∶1) for 5 h. Cell were washed and incubated for 3 hours more and PS translocation (annexin V-PE staining) and membrane integrity (AAD staining), mitochondrial membrane potential (DiOC6(3)) and calreticulin expression on the cell membrane were analysed by FACS as described in materials and methods. (1.57 MB TIF) [file pone.0007512.s001.tif]

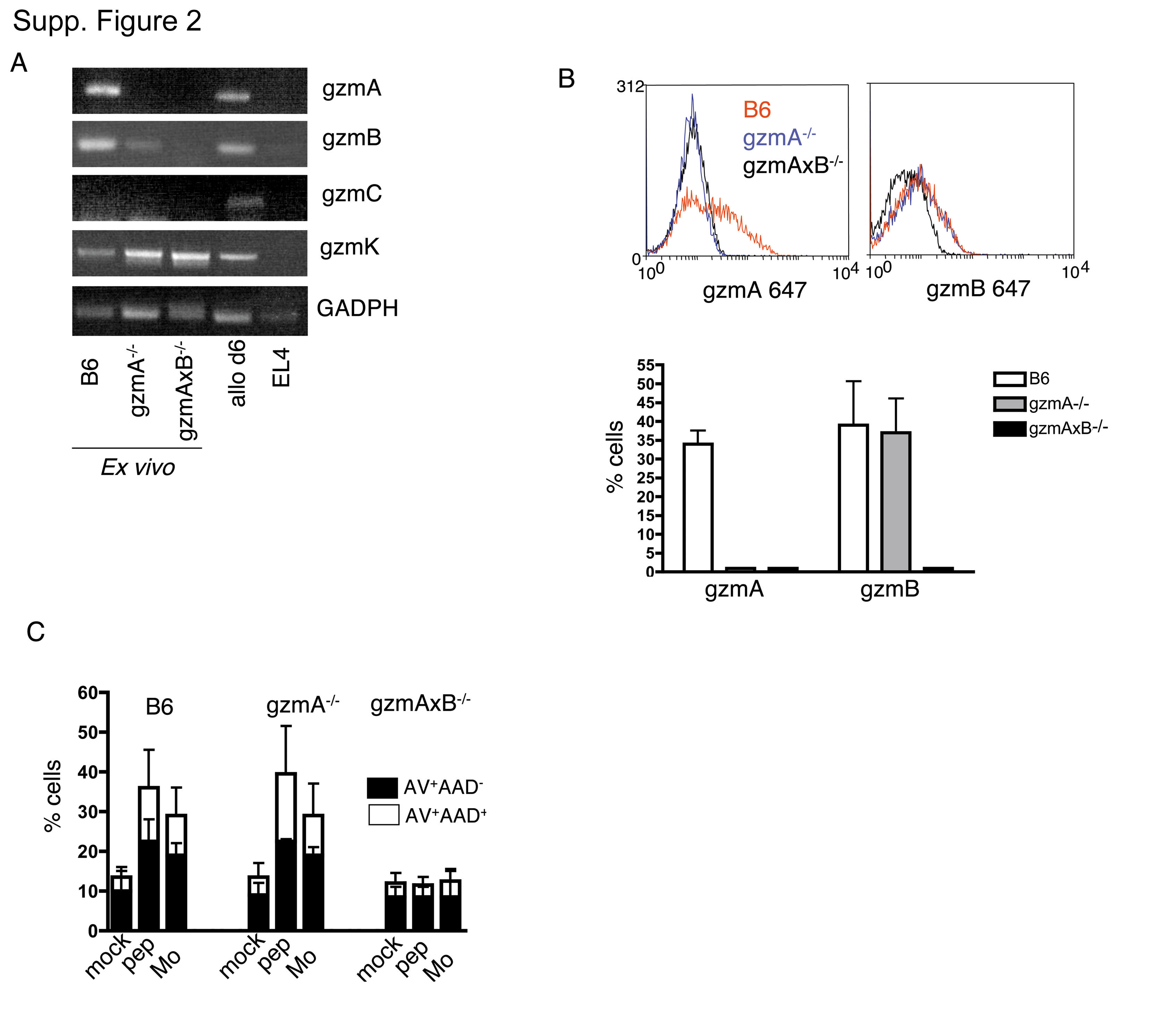

Supplement: Figure S2 — Characterization of ex vivo Tc cells from ECTV infected mice. CD8+ cells (anti-ECTV ex vivo Tc cells) were isolated by autoMACS from six-day HE-ECTV immune WT (B6), gzmA−/− or gzmA×B−/− mice. A, Total RNA was isolated and gzmA, gzmB, gzmC, and gzmK transcripts were analysed by RT-PCR. EL4 served as negative and day 6 primary allogeneic Tc cells as positive control. GADPH was analysed as housekeeping gene. B, intracellular gzmA and gzmB protein expression detected with anti-gzmA or anti-gzmB rabbit antiserum. Data shown are representative histograms of anti-gzm fluorescence (top panel) and means+/−SEM of percent gzm+ CD8+ cells (bottom panel) from 3 independent experiments. (2.07 MB TIF) [file pone.0007512.s002.tif]
